# 684 Implementation of an Interdisciplinary Scar Board

**DOI:** 10.1093/jbcr/iraf019.313

**Published:** 2025-04-01

**Authors:** Chelsea Gamero, Lisa McMurtrey, Irma Fleming, Christopher LaChapelle, Giavonni Lewis, Callie Thompson

**Affiliations:** University of Utah Health Burn Center; University of Utah Health Burn Center; University of Utah Burn Critical Care; University of Utah Health; University of Utah Health; University of Utah Health

## Abstract

**Introduction:**

Our Burn Center team provides comprehensive evaluation & management of burn scars, including laser treatment. Occasionally, it is necessary to involve the plastic surgery team for complex reconstructive procedures. As we developed our burn scar laser treatment program, we noted an opportunity to improve communication between teams. The leaders of these teams met to create the Scar Board (SB), modeled after interdisciplinary tumor boards. The SB was implemented with the following goals:

1) Improve communication between teams. 2) Improve coordination of care for patients requiring reconstructive surgery. 3) Increase the efficiency of placing referrals between teams.

SB includes members of the Burn MD/APC/RN teams, the Plastic Surgery MD team & Burn OT/PT staff.

**Methods:**

The burn team meets monthly to pre-screen patients that may benefit from a plastic surgery referral. Identified patients are presented bi-monthly at a SB meeting. Patient cases are presented & time is allowed for interdisciplinary discussion. Case presentations include pertinent patient information, images & or videos highlighting areas of concern. SB meetings began November 2020 & occur virtually allowing flexibility in attendance. Once patients are reviewed, referrals are placed & appointments are scheduled with the appropriate team. Referred patients are discussed at future SB meetings to address continued coordination of care. Members of the SB were given a survey to address satisfaction with the process. To date 177 patients have been pre-screened, 53 patient cases presented at interdisciplinary SB with 32 successful referrals to the plastic surgery team.

**Results:**

The survey found 87.5% of participants strongly agreed/agreed that instituting bi-monthly SB meetings improved communication between teams. One hundred percent of participants strongly agreed/agreed that SB meetings improved care of burn patients requiring reconstructive surgery. One hundred percent of participants strongly agreed/agreed that SB meetings increase efficiency of burn referrals to the plastic surgery team. When asked to rank satisfaction of SB meetings on a scale of 1-5 with one being very dissatisfied & five being very satisfied, the average rank was four.

**Conclusions:**

SB has shown to increase communication, improve care coordination & increase the efficiency of placing referrals. The survey results demonstrate that participants support this patient care model.

**Applicability of Research to Practice:**

This patient case review model can be utilized in other institutions to improve communication & collaboration between service teams.

**Funding for the Study:**

N/A

